# Serum fatty acid and lipoprotein subclass concentrations and their associations in prepubertal healthy Norwegian children

**DOI:** 10.1007/s11306-016-1020-y

**Published:** 2016-03-15

**Authors:** Tarja Rajalahti, Chenchen Lin, Svein Are Mjøs, Olav Martin Kvalheim

**Affiliations:** Fjordomics, Førde Hospital Trust, Førde, Norway; Department of Chemistry, University of Bergen, Bergen, Norway; Faculty of Health Studies, Sogn og Fjordane University College, Førde, Norway

**Keywords:** Human serum, Prepubertal healthy children, Lipoprotein subclasses, Fatty acids, Docosahexaenoic acid (DHA), Eicosapentaenoic acid (EPA)

## Abstract

**Introduction:**

The lipid metabolism is one of the most important and complex processes in the body. Serum concentrations of 18 fatty acids (FAs) and 24 lipoprotein features, i.e. concentrations of lipoprotein main and subclasses and average particle size in main classes, in 195 ethnic Norwegian children from the rural Fjord region were quantified by chromatography.

**Objectives:**

To assess gender differences in prepubertal children and reveal predictive FA patterns for lipoprotein features.

**Methods:**

Lipoprotein features were modelled from FA profiles using multivariate regression.

**Results:**

Contrary to observations for adults from the same region, gender differences in prepubertal children were generally small. However, higher concentrations of C16–C18 FAs for girls compared to boys correlated to higher concentrations of triglycerides (TG) and very low density lipoprotein (VLDL) particles and larger average size of VLDL particles. Concentrations of high density lipoprotein (HDL) and its subclass of medium particle size were higher in boys than in girls. These findings are opposite to observations in adults from the same region, but reflect that prepubertal boys are more physically active than girls. Furthermore, children possessed only half the serum levels of eicosapentaenoic acid and docosahexaenoic acid measured in adults. Since sampling was done after 12 h of fasting, these differences may reflect higher rate of utilization of these crucial FAs in children.

**Conclusion:**

Good predictive models were obtained for TGs, VLDL and chylomicrons with C14–C18 FAs as major contributors. Weak predictive associations were observed for HDL and Apolipoprotein A1 (ApoA1) with C20–C24 FAs as contributors.

**Electronic supplementary material:**

The online version of this article (doi:10.1007/s11306-016-1020-y) contains supplementary material, which is available to authorized users.

## Introduction

The lipid metabolism, whereby fatty acids are distributed by lipoproteins to build and maintain brain and body functions, is one of the most important and complex processes in the body. Serum lipoprotein patterns in humans are thus influenced by many factors. The pattern differs between genders and races (Freedman et al. 2000, 2001; Johnson et al. 2004) and changes occur during childhood and puberty (Stozicky et al. 1991; Labarthe et al. 2003; Dai et al. 2009). Cholesterol levels peak when children are 9–10 years old, then drop during puberty before increasing again during aging in adults (Freedman et al. 2004; Rajalahti et al. 2016). Lipoprotein levels are also impacted by diet and physical activity (Williams et al. 2005; Aadland et al. 2013), overweight (Pietiläinen et al. 2009), body fat (Spinneker et al. 2012) and diseases (Kuller et al. 2002). However, studies on populations with exceptional longevity (Barzilai et al. 2003) and studies on active and sedentary identical twins (Williams et al. 2005) show strong genetic control on many HDL and low density lipoprotein (LDL) features. Thus, ethnicity and gender imprint their signatures on an individual´s lipoprotein pattern but this is gradually modified through impact of aging and other factors.

In order to establish reference values and patterns for main and subclasses of lipoproteins, studies of healthy subjects are crucial. Based upon high performance liquid chromatography (HPLC) and curve fitting (Okazaki et al. 2005), Furusyo et al. (2013) established reference values for concentrations of 20 lipoprotein subclasses of cholesterol and triglyceride for Japanese men and women. The same approach was used by us to determine lipoprotein subclass concentrations in a cohort of healthy Norwegian adults (Lin et al. 2016). Furthermore, we quantified serum concentrations of the most biologically important fatty acids (FAs) and established multivariate models connecting lipoprotein features to predictive FA patterns in adults.

Our objectives with investigating levels of serum FAs and lipoprotein subclasses in healthy prepubertal children are: (i) to determine reference values and normal patterns for children that are detailed enough to be useful for detecting metabolic abnormalities, (ii) to disclose possible gender differences at pre-puberty, and, (iii) to reveal the strength and patterns of associations between FAs and lipoproteins and similarities and differences in the patterns for children and adults. To our knowledge, our study is the first to analyze subclass lipoprotein patterns in children and to quantify its association to FA patterns.

Univariate tests (Wilcoxon 1945) corrected for multiple testing (Benjamini and Hochberg 1995), hierarchical clustering analysis (HCA) (Kaufman and Rousseeuw 1990), multivariate discriminant (Sjöström et al. 1986; Rajalahti et al. 2010) and regression analysis (Wold et al. 1984) with post-processing by target projection (Kvalheim and Karstang 1989) and selectivity ratio (SR) plots (Rajalahti et al. 2009a, b) are used as tools to search for patterns.

## Materials and methods

### Participants

A cohort of 10 years old healthy ethnic Norwegian children, 117 boys and 78 girls, was recruited in the rural Fjord region of Western Norway. The children were recruited at age 10 in three rounds; 2011 (44 subjects), 2012 (49 subjects), and, 2014 (102 subjects).

### Blood sampling

Samples were collected between 8 and 10 am after overnight fasting. Serum was obtained according to the standardized protocol described in Lin et al. (2016), split into 0.5 ml aliquots, and stored in cryo tubes at −80 °C. At this temperature, the compound classes that are analyzed, i.e. lipoproteins and fatty acids, are stable for several years (Hodson et al. 2002; Jansen et al. 2014).

### Quantification of fatty acids

Serum samples were prepared and 18 FAs were quantified (Table 1) using a standardized protocol based on chromatographic analysis as described in Lin et al. (2016). The samples from the three rounds of sampling were randomized before analysis. Total amounts of FAs in each sample were converted to amounts in μg per g sample by dividing with the sample weights. This dataset includes the majority of FAs that are considered biologically important. Total fatty acid (TFA) concentration and the ratio of EPA to arachidonic (AA), i.e. EPA/AA, were also calculated (Table 1). Systematic names and abbreviations to common names defined in text are used. Supplementary material 1 contains ChEBI ids.

**Table 1:** Univariate statistical measures calculated for fatty acids for the pre-puberty children

Variable	Boys (N = 117)			Girls (N = 78)			p_WMW_
	Median	Min	Max	Median	Min	Max	
14:0	36.0	15.0	134.7	37.6	13.9	83.6	0.0966
16:0	764.6	432.3	1203.2	804.3	472.1	1359.1	0.1623
16:1 n-9	12.8	6.5	25.1	14.5	6.7	26.8	0.0237
16:1 n-7	65.3	24.1	154.9	74.8	25.3	164.4	0.0401
18:0	300.0	156.1	524.7	307.3	187.4	537.1	0.3862
18:1 n-9	731.9	442.1	1362.3	776.2	351.3	1317.7	0.1631
18:1 n-7	45.9	28.1	78.4	50.5	26.6	86.4	0.0025**
18:2 n-6 (LA)	1061.3	559.1	1767.9	1094.0	683.3	1837.3	0.2571
18:3 n-3 (ALA)	23.6	9.4	58.9	25.2	11.2	60.0	0.1466
20:3 n-6 (DGLA)	61.7	26.8	101.5	62.0	30.7	110.2	0.9866
20:4 n-6 (AA)	244.4	113.8	445.7	247.9	127.5	430.7	0.9370
22:0	33.9	15.3	62.9	35.5	21.2	66.1	0.1126
20:5 n-3 (EPA)	29.3	9.1	128.9	29.3	13.3	89.8	0.8714
24:0	32.0	12.9	58.4	32.8	20.1	61.9	0.4348
22:5 n-6	5.3	2.3	10.7	5.4	1.6	11.5	0.4900
24:1 n-9	52.7	21.3	97.3	54.7	32.8	106.5	0.0676
22:5 n-3 (DPA)	28.3	17.3	45.6	28.4	15.4	44.1	0.7807
22:6 n-3 (DHA)	73.8	34.6	153.5	79.4	35.3	206.6	0.3504
TFA	3610.9	2014.1	5730.8	3896.0	2151.9	5765.2	0.1702
EPA/AA	0.116	0.044	0.386	0.117	0.047	0.315	0.9535

Median, min and max values are given in units of μg per g sample. The column headed by p_WMW_ provides the p values calculated from the nonparametric Wilcoxon–Mann–Whitney (WMW) rank sum test (Wilcoxon 1945; Mann and Whitney 1947). Taking into account the effect of multiple testing on the probability levels show that p_WMW_ = 0.0025 corresponds to p = 0.05 whether the Bonferroni correction or the false discovery rate (FDR) of Benjamini and Hochberg (1995) is used. Gender differences that are significant at p = 0.05 after correcting for multiple testing are marked with two asterisks

### Quantification of lipoproteins

Serum lipoproteins were analyzed on an HPLC system at Skylight Biotech (Akita, Japan) and quantification for the main lipoproteins and 20 lipoprotein subclasses obtained according to the procedure described by Okazaki et al. (2005). The analyses were done in three batches corresponding to the three rounds of sample collection. Each batch was analyzed 2–3 months after sampling. In order to be able to reveal and correct for possible systematic analytical differences, five randomly selected samples from the first round were included and reanalyzed in the second and third round.

Serum apolipoproteins A1 and B were measured by turbidimetric immunoassay using commercially available kits (Sekisui Medical co., Ltd, Tokyo, Japan) for the samples collected in the first and second round (93 subjects).

### Merging of lipoprotein subclasses

Using the procedure described in Lin et al. (2016), the following lipoprotein features (Table 2) were calculated: Concentrations of total cholesterol (Chol) and total triglyceride (TG), total concentrations of chylomicrons (CM), very low density lipoproteins (VLDL), low density lipoproteins (LDL) and high density lipoproteins (HDL) particles, concentrations of 4, 4 and 5 subclasses of VLDL, LDL and HDL particles, respectively, labeled as VLDL-VL, VLDL-L. VLDL-M, VLDL-S, LDL-L, LDL-M, LDL-S, LDL-VS, HDL-VL, HDL-L, HDL-M, HDL-S and HDL-VS. The abbreviations VL, L, M, S and VS denote very large, large, medium, small and very small particles, respectively. In addition, average size of VDL, LDL and HDL particles were estimated.

**Table 2:** Univariate statistical measures calculated for fatty acids for the pre-puberty children

Variable	Boys (N = 117)			Girls (N = 78)			p_WMW_
	Median	Min	Max	Median	Min	Max	
Chol	163.6	118.3	236.1	164.7	107.1	254.5	0.5829
TG	49.7	17.3	137.1	65.9	20.8	135.8	0.0083*
CM	0.64	0	10.9	1.12	0	14.8	0.0618
VLDL	44.0	14.0	141.2	59.9	10.4	123.5	0.0019**
LDL	99.1	63.5	149.9	100.2	51.1	180.6	0.3970
HDL	67.3	42.0	99.0	63.1	44.7	99.9	0.0057*
VLDL-VL	7.6	1.1	48.5	13.0	1.5	51.3	0.0062*
VLDL-L	11.5	0	42.1	19.1	0	43.2	0.0003**
VLDL-M	12.1	2.5	36.2	14.5	4.0	35.2	0.0130*
VLDL-S	13.0	6.5	22.3	13.5	7.1	24.4	0.3722
LDL-L	39.7	23.2	55.2	38.9	23.9	59.8	0.7619
LDL-M	46.1	25.8	69.0	46.8	25.7	84.2	0.3451
LDL-S	12.2	3.0	28.4	12.9	2.5	28.6	0.0824
LDL-VS	4.7	1.6	10.9	4.9	1.1	11.8	0.0646
HDL-VL	3.8	1.5	10.8	3.7	2.0	9.9	0.3778
HDL-L	16.0	3.3	38.0	13.9	5.2	38.3	0.1327
HDL-M	24.4	14.4	33.9	22.6	15.1	32.1	0.0010**
HDL-S	18.0	12.6	24.4	17.3	11.9	23.1	0.0556
HDL-VS	5.4	3.4	10.5	5.4	3.1	9.4	0.8137
VLDL-Size	41.3	36.0	47.1	43.0	37.7	48.2	0.0043*
LDL-Size	26.19	25.71	26.79	26.14	25.74	26.49	0.1123
HDL-Size	10.91	10.23	11.54	10.91	10.46	11.44	0.4079
ApoA1	138.0^a^	91.1	187.0	127.1^b^	106.1	175.0	0.0591
ApoB	66.4^a^	47.4	86.0	65.4^2^	49.0	107.0	0.5290

Median, min and max concentrations are given in units of mg per dl serum, while particle size is given as nm. p_WMW_ are the p values calculated from the nonparametric Wilcoxon-Mann–Whitney (WMW) rank sum test (Wilcoxon 1945; Mann and Whitney 1947). Taking into account the effect of multiple testing on the probability levels show that p_WMW_ = 0.0021 corresponds to p = 0.05 using the Bonferroni correction. Using instead the false discovery rate (FDR) of Benjamini and Hochberg (1995) for multiple testing, p_WMW_ = 0.0167 corresponds to p = 0.05. Gender differences that are significant at p = 0.05 after the Bonferroni correction are marked with two asterisks, while differences that are significant by FDR at p = 0.05 are marked with one asterisk

^a^N = 55

^b^N = 38

### Correcting lipoprotein profiles for systematic batch differences

**Fig. 1:**
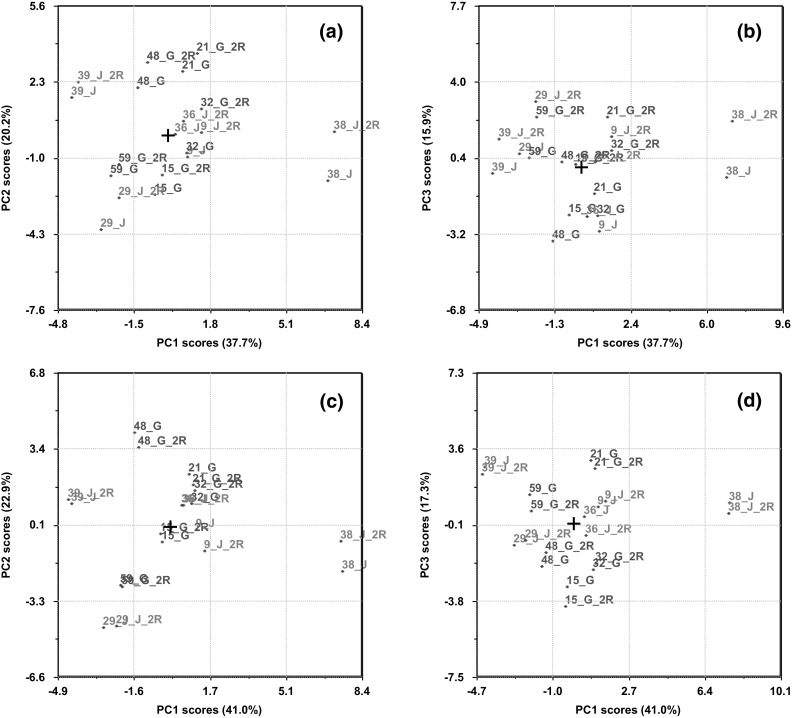
Principal component (PC) scores before (**a**, **b**) and after (**c**, **d**) the median difference correction (Rajalahti et al. 2016) to reduce systematic analytical differences between the three batches. Five samples from run 1 were reanalyzed in run 2 and 3. Pairs of replicates can be identified by possessing the same number with one replicate containing an R in the sample id

Principal component analysis (PCA) (Jolliffe 1986) of the five samples that were replicated in the second and third round of lipoprotein analysis revealed systematic differences between replicates. Figures 1a, b display the replicated samples on PC1 and PC2 and PC1 and PC3. The three PCs accounts for 73.8 % of the total sample variation. Pairs of replicates can be recognized by possessing the same number, but with one sample also having an R in its label. The score plots in Figs. 1a, b reveals a spread between replicates.

The lipoprotein features responsible for the systematic batch difference were detected using PLS-DA with batch no. as y-variable and then using the SR plot as described in Supplementary Material 2. Differences between the five replicates from the first and second batch of analyses were calculated for those features where the SR plot revealed systematic differences between the medians of the first and second batch. The measurements of the second batch were corrected by adding the median difference between the five replicates for each variable to all samples in batch 2. This procedure, called median difference correction (MDC) (Rajalahti et al. 2016), was repeated to correct systematic differences between the third and first batch. The merits of the correction was validated in three ways: (i) by doing a PLS-DA on the corrected data and inspect the SR plot (see Supplementary Material 2), (ii) by performing PCA of the replicates after MDC (Fig. 1c, d above) and visually inspect how close pairs of replicates coincided in the score plots on the major PCs, and, (iii) finally, by recalculating the models in Table 3 for the lipoprotein feature that was least explained (HDL-Size) and best explained (TG) based only on the last batch of 102 samples. For both features, the FA patterns turned out to be the same for the models based on all samples as for the models using only the samples from the last batch (not shown). Univariate measures for the lipoprotein features after adjustments for systematic batch differences are provided in Table 2.

**Table 3:** Lipoprotein features modelled from the fatty acid profiles, children (N = 195)

Variable	R2Y^a^	Q2Y^b^	FAs ranked after importance in model
Chol	0.36	0.26	DPA (0.45)^c^, DGLA, 18:1 n-7, 16:1 n-7, TFA, 16:0, AA (0.38)
TG	0.73	0.70	14:0 (0.61), 16:1 n-9, 16:1 n-7, 18:1 n-9, ALA (0.46)
CM	0.61	0.58	14:0 (0.65), 16:1 n-9, 16:1 n-7, 18:1 n-9, ALA (0.44)
VLDL	0.68	0.62	16:1 n-9 (0.62), 16:1 n-7, 18:1 n-9, 14:0, 18:1 n-7 (0.47)
LDL	0.34	0.21	18:1 n-7 (0.35), 16:1 n-7, TFA, 16:0, 18:1 n-9, DPA, LA (0.34)
HDL	0.16	0.08	24:0 (0.21), 22:0, AA (0.16)
VLDL-VL	0.67	0.64	14:0 (0.59), 16:1 n-9, 16:1 n-7, 18:1 n-9, ALA (0.43)
VLDL-L	0.63	0.58	16:1 n-7 (0.59), 16:1 n-9, 18:1 n-9, 18:1 n-7, 14:0, ALA (0.39)
VLDL-M	0.49	0.41	16:1 n-9 (0.52), 18:1 n-9, 18:1 n-7, 16:1 n-7, ALA (0.38)
VLDL-S	0.29	0.22	16:1 n-9 (0.46), 16:1 n-7, 16:0, 18:1 n-9, 18:1 n-7, 14:0, TFA (0.39)
LDL-L	0.16	0.12	TFA (0.39), 16:0, 18:0, 18:1 n-7, DPA, LA (0.34)
LDL-M	0.33	0.20	18:1 n-7 (0.31) 16:1 n-7, TFA, 18:1 n-9, 16:0, LA, DPA (0.30)
LDL-S	0.38	0.24	DPA (0.29), LA, 16:1 n-7, TFA, 18:1 n-7 (0.28)
LDL-VS	0.40	0.26	DPA (0.33), 16:1 n-7, LA, TFA, 18:1 n-7 (0.30)
HDL-VL	0.18	0.11	24:0 (0.26), 22:0, AA (0.16)
HDL-L	0.20	0.13	24:0 (0.19), 22:0, AA (0.12)
HDL-M	0.20	0.08	24:0 (0.17), 22:0, 18:0, 16:1 n-7 (-), 18:1 n-7 (-), AA (0.10)
HDL-S	0.36	0.28	16:1 n-9 (0.30), 14:0, DGLA, 18:1 n-9, 16:1 n-7 (0.27)
HDL-VS	0.21	0.09	DGLA (0.25), DPA, 16:1 n-9, 22:5 n-6, 16:1 n-7 (0.18)
VLDL-size	0.53	0.49	14:0 (0.52), 16:1 n-9, 16:1 n-7, 18:1 n-9, ALA (0.32)
LDL-size	No predictive model		
HDL-size	0.22	0.15	14:0 (-0.24), 16:1 n-7 (-), 16:1 n-9 (-0.24)
ApoA1	0.30	0.18	24:0 (0.27), ALA (-), AA, 22:0 (0.23)
ApoB	0.15	0.08	Tot FA (0.35), 16:0, 18:0, DGLA (0.28)

^a^R2Y implies the squared Pearson correlation coefficient between measured and modelled lipoprotein feature

^b^Q2Y implies the squared Pearson correlation coefficient between measured and independently predicted values for the lipoprotein feature using RDCV (Westerhuis et al. 2008). Q2Y is calculated as the average of 100 repetitions with 10 % of ssamples in outer loop

^c^Pearson´s correlation coefficient estimated from the raw data, see Supplementary Material 3

### Data analysis

Normal probability plots of FAs and lipoprotein features revealed that many variables were far from normally distributed. The non-parametric Wilcoxon-Mann–Whitney (WMW) unpaired rank sum test (Wilcoxon 1945; Mann and Whitney 1947) was therefore used to test the null hypothesis of equal medians for the 20 FA (Table 1) and 24 lipoprotein (Table 2) features. Calculations were performed using Matlab R2013b (MathWorks, Natick, MA, USA). Assuming that the 20 FA features constitute one family of test and the 24 lipoprotein features constitute a second family of tests, the p values in Table 1 and 2 have to be corrected for multiple testing. This correction can be done in different ways: (i) Using the Bonferroni approach and multiply each *p* value by the number of variables in a family to obtain the corrected p value, or, (ii) using the concept of false discovery rate (FDR) (Benjamini and Hochberg 1995) to reveal the variables with significantly differing medians. While the Bonferroni correction may lead to acceptance of the null hypothesis of identical medians too often and thus, a tendency to overlook true gender differences, FDR may lead to rejection of the null hypothesis too often and thus, an increased tendency to detect false gender differences. Therefore we provide the results of both corrections to identify borderline cases.

Cross correlations between lipoprotein features and FAs in children were determined as Pearson´s correlation coefficients (Supplementary Material 3) and the FA correlation patterns used as input to agglomerative HCA of lipoprotein features with Euclidean distance as metric and average linkage for clustering (Kaufman and Rousseeuw 1990).

Multivariate data analysis was performed by means of the commercial software Sirius Version 10.0 (Pattern Recognition Systems AS, Bergen, Norway). Prior to multivariate analysis, all variables were centered and standardized to unit variance. Partial-least squares discriminant analysis (PLS-DA) (Sjöström et al. 1986, Rajalahti et al. 2010) was used to test FA and lipoprotein profiles for possible discriminating patterns between boys and girls. The FAs (Table 1) and lipoprotein features (Table 2) were modelled separately. Repeated double cross validation (RDCV) (Westerhuis et al. 2008) with 100 repetitions, 10 % of subjects in outer loop and 1/7 of remaining subjects kept out iteratively in inner loop was used for optimizing the predictive performance of the models. The validation showed that neither the FAs nor the lipoprotein profiles possessed predictive multivariate patterns discriminating boys and girls.

Standard PLS regression models (Wold et al. 1984) were created for all the 24 lipoprotein features in Table 2 with the FAs in Table 1 as input. Due to small differences in lipoprotein features and FA profiles for children revealed by the univariate tests, boys and girls were modelled jointly. PLS models were built using the same procedure as for the PLS-DA models. Q2Y was used to determine the dimension of the models (Westerhuis et al. 2008), but to reduce the problem with overfitting in PLS (Cloarec 2014), we used a stronger criterion than just the maximum Q2Y for model selection. For a sufficient number of PLS components to encompass the maximum value of Q2Y, the mean and the standard deviation around the mean were calculated from the 100 repeated runs using RDCV. The number of PLS components providing the model with optimal predictive power was determined by comparing Q2Y minus two standard deviations around the mean of component *a* + *1* with the mean of Q2Y for the previous component *a* starting with *a* = 1 and continuing with *a* = 2 etc. until a component *a* + *1* is found with its the mean Q2Y reduced by two standard deviations being lower or equal to the mean of Q2Y for component *a*. The optimal number of PLS components is then determined as *a* (Rajalahti et al. 2016). The models obtained by RDCV were further validated by randomization using 1000 permutations with 10 % of subjects in outer loop. All models had p < 0.005. For each cross-validated PLS model, a single predictive component was subsequently calculated by means of target projection (TP) (Kvalheim and Karstang 1989; Rajalahti and Kvalheim 2011). SRs were obtained as the ratio of explained to residual variance for each FA on the predictive TP component (Rajalahti et al. 2009a, b). The ratios are displayed in an SR plot with plus or minus sign indicating positive or negative correlation, respectively, with the lipoprotein feature modelled. The sign for each SR is determined from the corresponding loading on the predictive TP component. The SR plots display the FAs according to their discriminatory importance for each predicted lipoprotein feature and were used for ranking the FAs in each of the 24 models. Due to the cross validation procedure, RDCV with 100 repetitions and 10 % of samples in outer loop, confidence bounds can be constructed around each SR value and used as a check of the significance of the ratio for each variable. Limits corresponding to two standard deviations are displayed in the SR plots.

## Results and discussions

### Gender differences in FA and lipoprotein features

From Table 1, we observe small gender differences in median levels for the individual FAs for children using Wilcoxon–Mann–Whitney (WMW) rank sum test (Wilcoxon 1945; Mann and Whitney 1947). Although the medians of most FAs are lower in in boys than in girls, only the 18:1 n-7 is significantly lower at p = 0.05, corresponding to p_WMW_ = 0.0025, using either the Bonferroni correction for multiple testing or the false discovery rate (FDR) of Benjamini and Hochberg (1995).

Also the lipoprotein features are similar in pre-puberty children (Table 2). Significance level of 0.05 for Bonferroni corrected p-values corresponds to p_WMW_ = 0.0021. Only three lipoprotein features are significantly different at p = 0.05 after Bonferroni correction for multiple testing: Total concentrations of VLDL particles (p = 0.046), and the concentrations of the subclasses VLDL-L (p = 0.007) and HDL-M (p = 0.024). If we instead apply the FDR approach, the limit of significance corresponding to p = 0.05 is p_WMW_ = 0.0167 and also the median concentrations of TG, HDL, the subclasses VLDL-L and VLDL-M, and the average size of VLDL particles (VLDL-size), show gender differences. All the lipoprotein features connected to VLDL particles, except concentration of VLDL-S, are higher in girls than boys in our cohort. Also HDL and the subclass HDL-M have higher medians in boys than in girls. These findings are contrary to what is observed in adults (Lin et al. 2016). This may reflect that the boys are more physically active than the girls in the age group we are studying. Thus, a study of 9–10 years Utah children (Hager et al. 1995) showed lower level of TG and higher of HDL in boys than girls and these observations correlated to better physical fitness in boys as measured by maximal aerobic capacity (VO_2max_). Williams et al. (2005) compared pairs of active and sedentary identical twins and found significant higher levels of HDL for both genders in the physically active twins. Physical activity triggers reverse cholesterol transport (RCT) that increases concentrations of HDL particles, while TG and VLDL and their subclasses of very large and large particles decrease with increased physical activity (Aadland et al. 2013). Comparison of the levels of the standard lipid panel shows that 10 years old Norwegian children have almost the same level of total cholesterol as 11 years old American children (Dai et al. 2009), but considerably higher level of HDL cholesterol (57.6 vs. 51.2 mg/dL) and lower level of LDL cholesterol (82.8 vs. 94.8 and 91.8 mg/dL for boys and girls, respectively) and TG (54.1 vs. 85.8 mg/dL).

PLS-DA of the FA profiles and the lipoprotein features for children with gender as dependent variable (0 for boys, 1 for girls) gave no predictive PLS components. These results underline that pre-pubertal boys and girls have similar FA (Table 1) and lipoprotein (Table 2) patterns.

### Hierarchical clustering of lipoprotein features based on FA correlation patterns

**Fig. 2:**
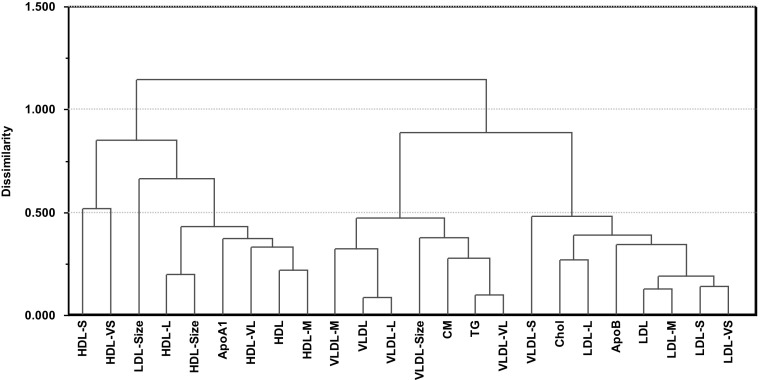
Dendrogram from agglomerative hierarchical cluster analysis calculated from average-linkage using Euclidean distance as metric. The dendrogram maps the correlation patterns (Supplementary Material 3) between each of the 24 lipoprotein feature (Table 2) and the 20 FA features (Table 1)

Due to the small differences in lipoprotein and FA patterns between boys and girls, the children were analyzed jointly by hierarchical clustering. Each lipoprotein was described by its correlations with the 20 FA features (Supplementary Material 3). Figure 2 displays the results.

A division into three main groups is evident. At the right side of the dendrogram, all the LDL subclasses link together with ApoB, Chol and the subclass of small VLDL particles. The latter is often denoted as intermediate density lipoprotein (IDL) subclass. In the middle of the dendrogram, all the other VLDL features cluster together with CM and TG. Finally, all the HDL features cluster in one group together with ApoA1 and average size of LDL particles.

Although the dendrogram overall is similar to what was found for a comparative cohort of adults (See Supplementary Material 4 from Lin et al. 2016), it reveals some important differences. Thus, the atherogenic subclasses LDL-S and LDL-VS (Hirayama and Miida 2012) grouped together with the VLDL features for men, and the possibly atherogenic subclasses HDL-S and HDL-VS (Lin et al. 2016 and refs. therein) grouped together with the VLDL features for both genders. For children, these subclasses cluster together with their main class lipoprotein implying that the FA patterns for these subclasses are similar to their “parent” lipoproteins, HDL and LDL, in healthy children. Note, however, that the subclasses of small and very small HDL are the last ones to be linked in the group of HDL features. Separate clustering of boys and girls (not shown) gave the same dendrogram for girls as from the joint clustering and only minor changes for boys. Thus, the subclass HDL-S grouped together with the VLDL features and the subclass HDL-VS grouped together with the LDL features for boys.

### Predicting lipoprotein features from FA profiles

In order to assess the strength of associations between FAs and lipoprotein features in children, we built validated regression model for each lipoprotein feature. Since the gender differences for prepubertal children appear to be small, both genders were modelled jointly.

Table 3 summarizes the results for the 24 lipoprotein models with strength of association measured by R2Y (the squared correlation between measured and predicted y-s when the predicted samples are also used to build the model) and predictive power by Q2Y (the squared correlation between measured and predicted y-s when the predicted samples are not used to build the model). The most strongly contributing FAs for each model are ranked according to their SR on the predictive component. For each lipoprotein model, Pearson correlation coefficients for the most predictive FAs are also provided in parentheses for the first and last FA in the list.

Strong predictive associations are observed for TG, concentration of VLDL, CM and the subclasses VLDL-VL and VLDL-L. They are all connected to C14–C18 saturated and monounsaturated FAs just as for the corresponding models for adults (Lin et al. 2016). LDL, all the subclasses of LDL, VLDL-M, VLDL-S, HDL-S and HDL-VS have moderate predictive associations to the FA profile with strongest contributions from C16–C18 mono-unsaturated FAs, DPA, linoleic acid (LA) and total concentration of FAs (TFA). The models for VLDL-M, VLDL-S, LDL-L, HDL-S and HDL-VS have predictive FA patterns that are similar to those observed in adults (Lin et al. 2016). Also ApoB and average size of VLDL particles show similar FA patterns in children as in adults. C20–C24 saturated FAs and arachidonic acid (AA) have weak predictive associations to ApoA1 and the subclasses HDL-VL, HDL-L, and HDL-M. Contrary to what was found for adults (Lin et al. 2016) the marine omega-3 FAs (EPA and DHA) seem unassociated to all of the lipoprotein features. For women, EPA and EPA/AA dominated the FA pattern for HDL-VL, HDL-L and average size of HDL particles, while for men the same features dominated the average size of LDL and HDL particles.

The lack of associations between lipoproteins and EPA and EPA/AA in children could mean, however, that these two crucial FAs are utilized at a higher rate during childhood due to high demand connected to development of brain and body. This may explain why they are low in children´s serum after 12 h of fasting. Rise et al. (2013) reported increase in *relative* levels of serum EPA and DHA from children to grown-ups for both genders. Rajalahti et al. (2016) found significant increase in EPA, DHA and docosapentaenoic acid (DPA) levels for both genders with aging.

An alternative explanation to the differences in levels of EPA and DHA in children and adults is that the dietary intake of the children is very different from the adults being separated by, on the average, 30 years in age. Since both children and adults live in the same Fjord region and are influenced by the same food culture, this seems less plausible than the biological hypothesis of explaining the difference as growth (children) versus maintenance (adults) of the brain and body.

**Fig. 3:**
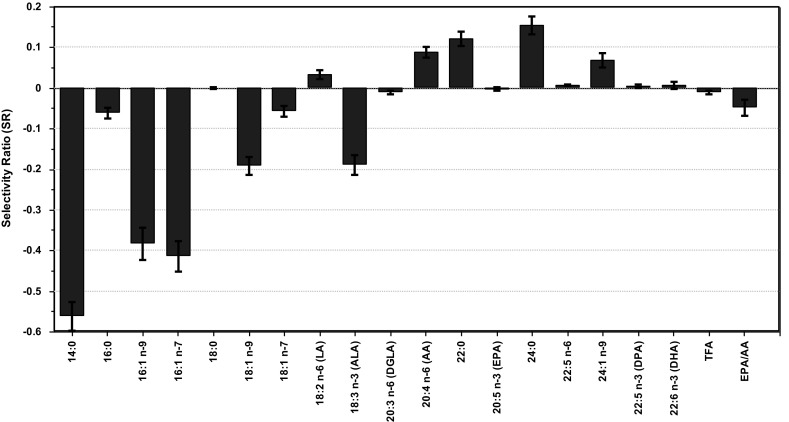
Selectivity ratio (SR) plot for model of average size of HDL particles (HDL-Size) with the 18 FAs, TFA and EPA/AA in Table 1 as input. Features with positive sign are increasing with HDL-size, while features with negative sign are decreasing. The confidence limits around each feature correspond to p = 0.05 and is obtained from RDCV

In order to substantiate our hypothesis that the lack of association between lipoprotein models and FAs in children is due to fasting, the SR plot for HDL-size is displayed (Fig. 3).

For both genders, EPA and EPA/AA were the two major contributors to the HDL-size model for adults. Figure 3 shows that, for children, these features have no predictive associations at all to HDL-Size. Furthermore, Fig. 3 shows that DHA apparently also has no impact on HDL-Size for children. It is unlikely that the marine omega-3 FAs should have large impact on adults, but no impact on children so this result strengthens the hypothesis that the apparent lack of associations of EPA and DHA to the HDL features is caused by fasting.

## Concluding remarks

We have shown that gender differences in pre-pubertal children are small both with respect to FA profiles and lipoprotein features. The girls have higher concentrations of TG and VLDL and its subclasses. These observations probably reflect lower level of physical activity for girls than boys in our age group and may be different in other cohorts.

For many features, the results for children differ from what was found for adults from the same region. Adults show large gender differences in lipoprotein features with men exhibiting a more atherogenic pattern than women (Lin et al. 2016) that is not observed in prepubertal children. The absolute concentrations of serum EPA and DHA are significantly lower in pre-pubertal children than in a matching cohort of adults and show no associations to the HDL subclasses as observed for adults, but this might be a result of overnight fasting.

## Electronic supplementary material

Below is the link to the electronic supplementary material.

Supplementary material 1 (ODS 48 kb)

Supplementary material 2 (PDF 96 kb)

Supplementary material 3 (XLSX 13 kb)

Supplementary material 4 (PDF 9 kb)
