# Supplementary material for: Serum fatty acid and lipoprotein subclass concentrations and their associations in prepubertal healthy Norwegian children
Source: Metabolomics. 2016 Mar 15;12:81. doi: 10.1007/s11306-016-1020-y (PMC4792365; doi:10.1007/s11306-016-1020-y)
Supplement: Supplementary file 2 — Supplementary material 2 (PDF 96 kb) [file 11306_2016_1020_MOESM2_ESM.pdf]

Supplementary material 2. SR plot to identify systematic batch differences and to validate successful batch correction.

The lipoprotein analyses were performed in three runs with the second and third batch of samples being analyzed approximately one and two years, respectively, after the analysis of the first batch. In order to be able to adjust for possible systematic analytical differences between the batches, five samples from the first batch were reanalyzed together with the samples from the second batch and similarly for the third batch. The lipoprotein profiles of these replicated samples were modelled using partial least squares discriminant analysis (PLS-DA) (Sjöström et al. 1986) with the y-variable having values 0 for the analyses in the first run and values 1 for the second run. Repeated double cross validation (RDCV) (Westerhuis et al. 2008) gave a model with 2 PLS components and R2Y=0.91 and with Q2Y=0.70. Selectivity ratios (SRs) (Rajalahti et al. 2009a; Rajalahti et al. 2009b) for the lipoproteins were calculated as explained in section 2.7 of the main document and revealed systematic differences in some lipoprotein features between the two batches (Fig. 1).

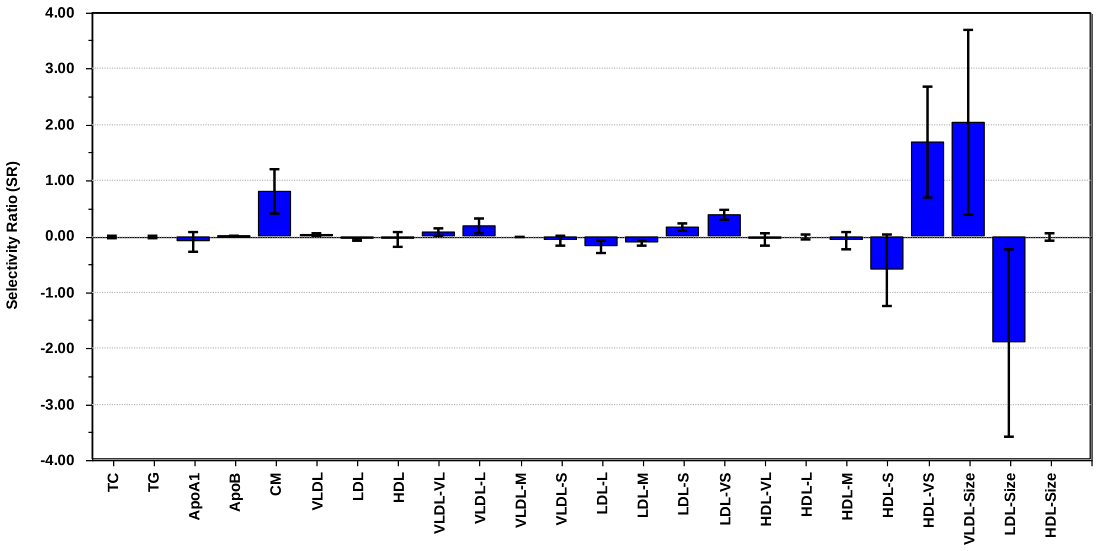

Figure 1. SR plot with confidence bounds of two standard deviations around mean.

The concentrations of LDL subclasses show a systematic shift towards smaller particle size and this is reflected in reduced average size of LDL particles. An opposite shift is observed in VLDL subclasses leading to larger increased average size of VLDL particles. A shift from small to very small HDL particles and increase in CM concentration is also evident in Fig. 1. For each of the lipoprotein features showing systematic differences, the batch difference for each replicate was calculated and the measurements of all samples in the second batch were adjusted by adding the median of these differences. This procedure assumes that the five replicates are representative for the analytical differences between batches and that the effect is additive. After this preprocessing, termed median difference correction (MDC), PLS-DA of the replicates gave no significant components and thus, the systematic differences had been removed. This can be confirmed by displaying the SR plot of the overfitted model with 5 PLS components which has R2Y=0.93. This is approximately the same as before batch correction.

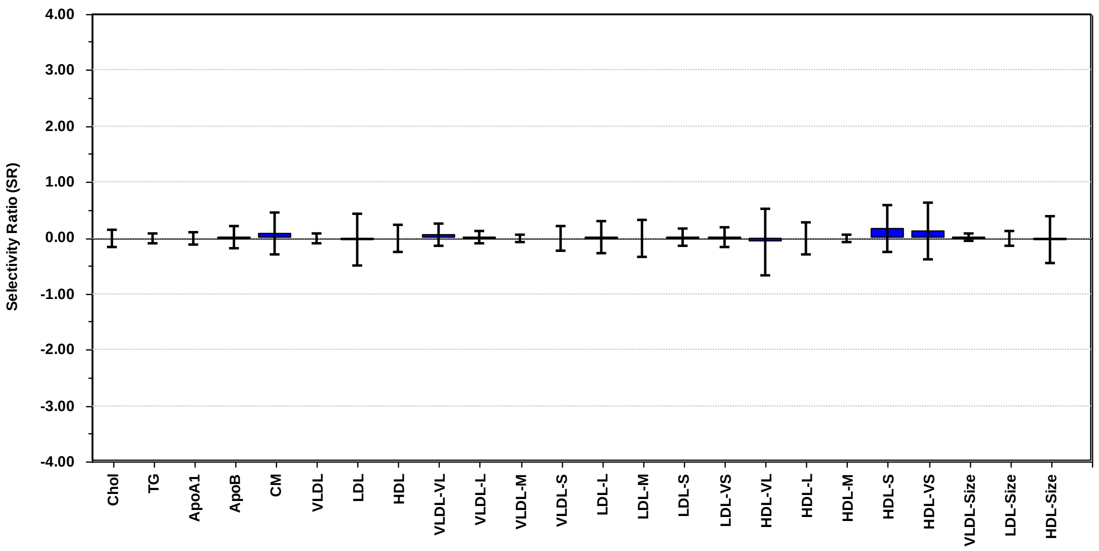

Figure 2. SR plot after median difference correction of batch 2.

Q2Y is strongly negative for this model since the predictive variation was exhausted with the batch correction.

The same approach was used to model and correct batch 3 to batch 1. R2Y and Q2Y was 0.98 and 0.80 before correction. Fig. 3 shows the SR plot before correction:

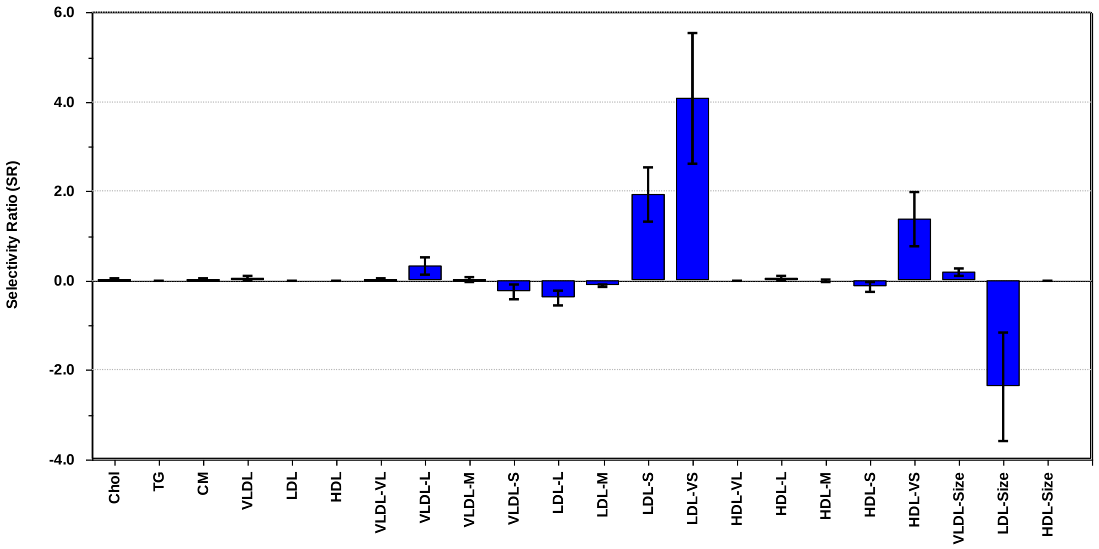

Figure 3. SR plot for batch 1 and 3 with batch no. as y-variable in PLS-DA.

A systematic shift is observed from large to small LDL particles which cause reduced average size of LDL particles. An opposite trend is observed for VLDL with increase from small to large particles leading to a small increase in average size of VLDL particles. Increase in very small HDL particles is also observed.

After batch correction, no predictive PLS-DA model is possible, but an overfitted 5 component model with R2Y=0.89 and Q2y less than zero provides the SR plot shown in Fig.4 and we conclude that the correction was successful.

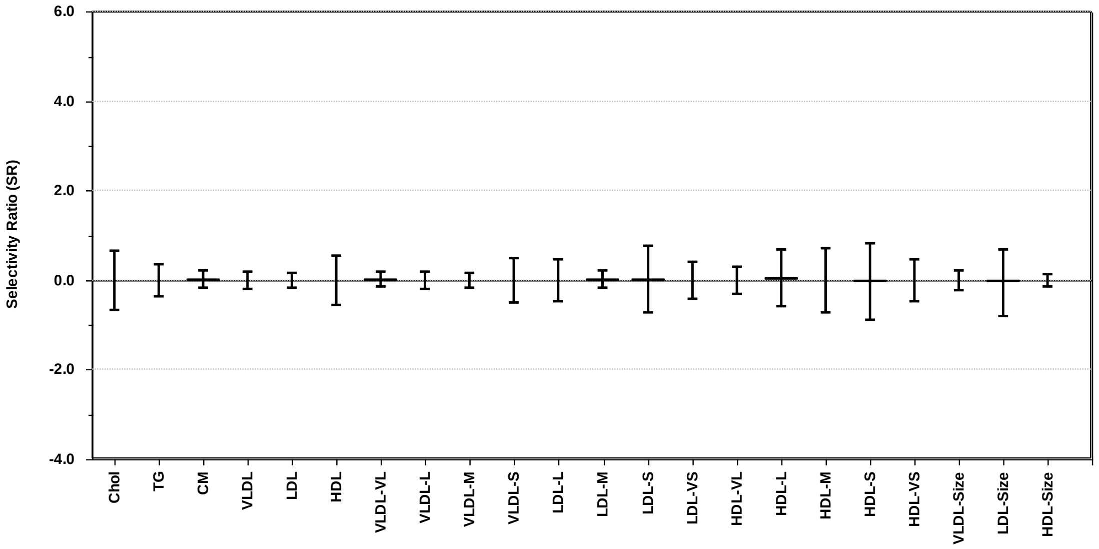

Figure 4. SR plot after median difference correction of batch 3.
