# Supplementary material for: Serum fatty acid and lipoprotein subclass concentrations and their associations in prepubertal healthy Norwegian children
Source: Metabolomics. 2016 Mar 15;12:81. doi: 10.1007/s11306-016-1020-y (PMC4792365; doi:10.1007/s11306-016-1020-y)
Supplement: Supplementary file 4 — Supplementary material 4 (PDF 9 kb) [file 11306_2016_1020_MOESM4_ESM.pdf]

Supplementary material 4. Agglomerative hierarchical cluster analysis (HCA) of lipoproteins.

Using HCA, the similarity of the lipoprotein features for both genders based on their fatty acid (FA) correlation patterns was assessed and presented as dendrograms for men and women (Lin et. 2016). In order to make the dendrograms available for comparison with the analogous results for the children in the main document, the dendrograms are displayed below

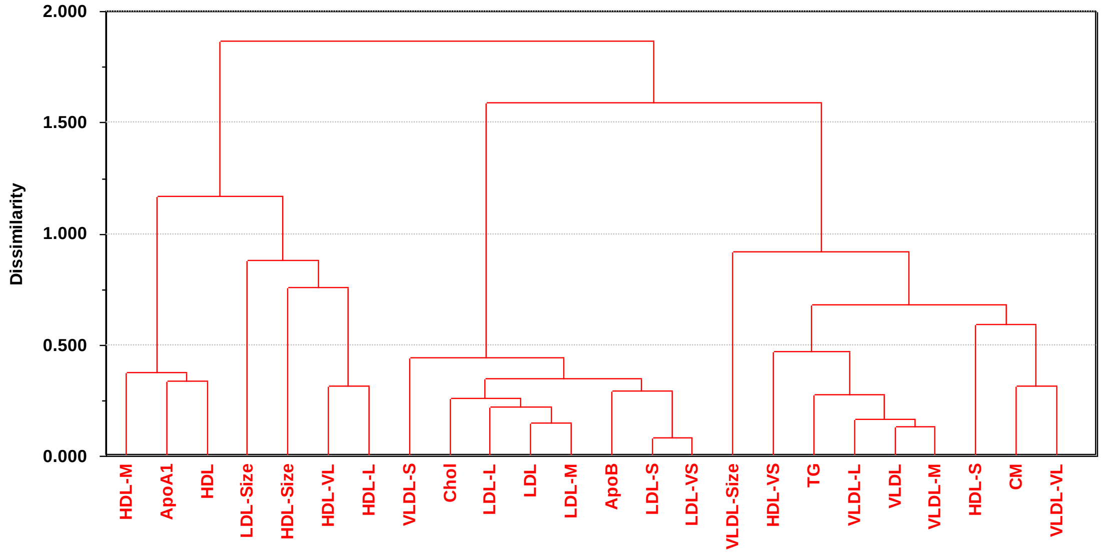

Figure 1. Dendrogram for women (from Lin et al. 2016).

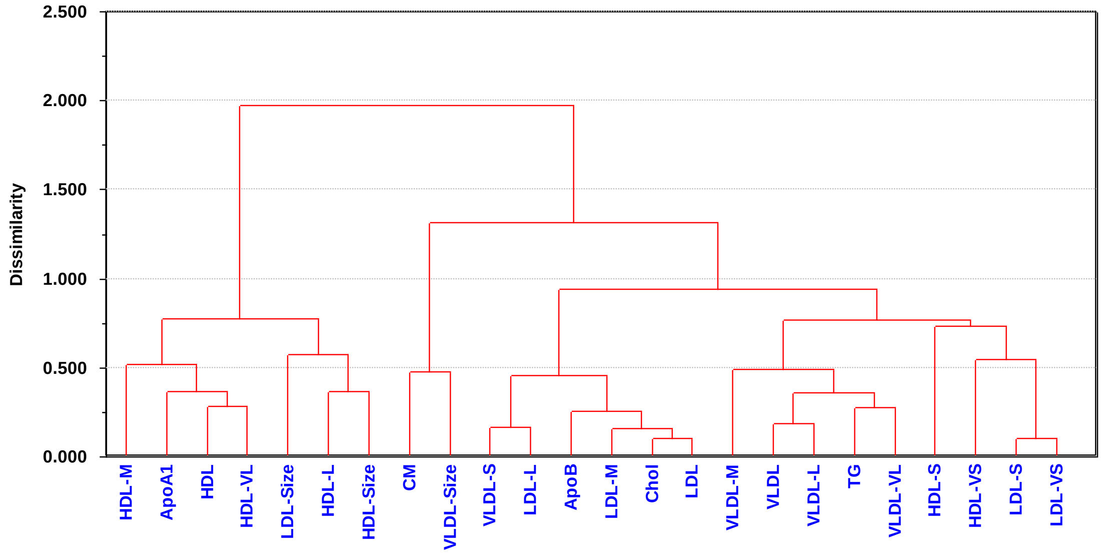

Figure 2. Dendrogram for men (from Lin et al. 2016).
